# Autologous haematopoietic stem cell transplantation and other cellular therapy in multiple sclerosis and immune-mediated neurological diseases: updated guidelines and recommendations from the EBMT Autoimmune Diseases Working Party (ADWP) and the Joint Accreditation Committee of EBMT and ISCT (JACIE)

**DOI:** 10.1038/s41409-019-0684-0

**Published:** 2019-09-26

**Authors:** Basil Sharrack, Riccardo Saccardi, Tobias Alexander, Manuela Badoglio, Joachim Burman, Dominique Farge, Raffaella Greco, Helen Jessop, Majid Kazmi, Kirill Kirgizov, Myriam Labopin, Gianluigi Mancardi, Roland Martin, John Moore, Paolo A. Muraro, Montserrat Rovira, Maria Pia Sormani, John A. Snowden, for the European Society for Blood and Marrow Transplantation (EBMT) Autoimmune Diseases Working Party (ADWP) and the Joint Accreditation Committee of the International Society for Cellular Therapy (ISCT) and EBMT (JACIE), John Snowden, Riccardo Saccardi, Eoin McGrath, Franco Bambi, Fermín Sanchez-Guijo, Nina Worel, John Snowden, Tobias Alexander, Manuela Badolglio, Mario Abinun, Renate Arnold, Charlotte Brierley, Joachim Burman, Cristina Castilla-Llorente, Nichola Cooper, Thomas Daikeler, Nicoletta del Papa, Dominique Farge, Jurgen Finke, Raffaella Greco, Hans Hagglund, Joerg Henes, Falk Hiepe, Helen Jessop, David Kiely, Myriam Labopin, Majid Kazmi, Kirill Kirgizov, Gianluigi Mancardi, Zora Marjanovic, Roland Martin, Thierry Martin, David Ma, John Moore, Paul Miller, Paolo Muraro, Maria-Carolina Oliveira, Alexey Polushin, Francesco Onida, Belinda Simoes, Mathieu Puyade, Igor Resnick, Montserrat Rovira, Riccardo Saccardi, Muhammad Saif, Ioanna Sakellari, Basil Sharrack, Emilian Snarski, Hans Ulrich Scherer, Claudia Sossa, Jeska de Vries-Bouwstra, Nico Wulffraat, Eleanora Zaccara

**Affiliations:** 1 Department of Neurology, Sheffield Teaching Hospitals NHS Foundation Trust, Sheffield, UK; 2 NIHR Neurosciences Biomedical Research Centre, University of Sheffield, Sheffield, UK; 3 Cell Therapy and Transfusion Medicine Unit, Careggi University Hospital, Firenze, Italy; 4 Klinik fur Rheumatologie und Klinische Immunologie, Charite-Universitatsmedizin, Berlin, Germany; 5 EBMT Paris study office, Department of Haematology, Saint Antoine Hospital, INSERM UMR 938, Sorbonne University, Paris, France; 6 Department of Neuroscience, Uppsala University, Uppsala, Sweden; 7 Unité de Médecine Interne, Maladies Auto-immunes et Pathologie Vasculaire (UF 04), Hôpital St-Louis, AP-HP, Paris, France; 8 Centre de Référence des Maladies Auto-Immunes Systémiques Rares d’Ile-de-France, Filière, FAI2R Paris, France; 9 EA 3518, Université Denis Diderot, Paris, France; 10 Department of Internal Medicine, McGill University, Montreal, QC Canada; 11 Hematology and Bone Marrow Transplantation Unit, Istituto di Ricovero e Cura a Carattere Scientifico, San Raffaele Scientific Institute, Milan, Italy; 12 Department of Haematology, Sheffield Teaching Hospitals NHS Foundation Trust, Sheffield, UK; 13 Kings Health Partners, Department of Haematology, Guys Hospital, London, UK; 14 N.N. Blokhin National Medical Center of Oncology, Institute of Pediatric Oncology and Hematology, Moscow, Russia; 15 Department of Neuroscience, University of Genova and Clinical Scientific Institutes Maugeri, Genoa, Italy; 16 Neuroimmunology and MS Research, Neurology Clinic, University Hospital, Zurich, Switzerland; 17 Haematology Department, St. Vincent’s Health Network, Darlinghurst, NSW Australia; 18 Department of Brain Sciences, Imperial College London, London, UK; 19 BMT Unit, Department of Hematology, IDIBAPS, Hospital Clinic, Institut Josep Carreras, Barcelona, Spain; 20 Department of Health Sciences (DISSAL), University of Genoa, Genoa, Italy; 21 IRCCS Ospedale Policlinico San Martino, Genoa, Italy; 22 Department of Haematology, Sheffield Teaching Hospitals NHS Foundation Trust, Sheffield, UK; 23 Haematology Department, Careggi University Hospital, Florence, Italy; 24 EBMT Executive Office, Barcelona, Spain; 25 Ospedale Pediatrico, Azienda Ospedaliero Universitaria Meyer (A.O.U. Meyer), Florence, Italy; 26 IBSAL, Hospital Universitario de Salamanca, Salamanca, Spain; 27 Blood Group Serology and Transfusion Medicine, Medical University of Vienna, Vienna, Austria; 28 Department of Haematology, Sheffield Teaching Hospitals NHS Foundation Trust, Sheffield, UK; 29 Klinik fur Rheumatologie und Klinische Immunologie, Charite-Universitatsmedizin, Berlin, Germany; 30 Great North Children’s Hospital, Institute of Cellular Medicine, Newcastle University, Newcastle upon Tyne, UK; 31 Department of Haematology, Oncology and Tumor Immunology, Charité Universitätsmedizin Berlin, Berlin, Germany; 32 Department of Haematology, Oxford University Hospitals NHS Foundation Trust, Oxford, UK; 33 Unité de transplantation des cellules souches, Département d’Hématologie Gustave Roussy, Villejuif, France; 34 Department of Haematology, Hammersmith Hospital, Imperial College Health Care NHS Trust, London, UK; 35 University and University Hospital of Basel, Department of Rheumatology, Basel, Switzerland; 36 Osp. G. Pini, Department of Rheumatology, Milan, Italy; 37 Unité de Médecine Interne: Maladies Auto-immunes et Pathologie Vasculaire (UF 04), Hôpital St-Louis, AP-HP, Paris, France; 38 Centre de Référence des Maladies Auto-Immunes Systémiques Rares d’Ile-de-France, Filière FAI2R, Paris, France; 39 EA 3518, Université Denis Diderot, Paris, France; 40 Department of Medicine-Hematology and Oncology, University of Freiburg, Freiburg, Germany; 41 Hematology and Bone Marrow Transplantation Unit, Istituto di Ricovero e Cura a Carattere Scientifico, San Raffaele Scientific Institute, Milan, Italy; 42 Department of Hematology, Uppsala University Hospital, Uppsala, Sweden; 43 Centre for Interdisciplinary Clinical Immunology, Rheumatology and Auto-inflammatory Diseases, University Hospital Tuebingen, Department of Internal Medicine II (Oncology, Haematology, Immunology, Rheumatology, Pulmonology), Tuebingen, Germany; 44 Klinik fur Rheumatologie und Klinische Immunologie, Charite-Universitatsmedizin, Berlin, Germany; 45 Department of Haematology, Sheffield Teaching Hospitals NHS Foundation Trust, Sheffield, UK; 46 Sheffield Pulmonary Vascular Disease Unit, Sheffield Teaching Hospitals NHS Foundation Trust, Sheffield, UK; 47 Kings Health Partners, Department of Haematology, Guys Hospital, London, UK; 48 N.N. Blokhin National Medical Center of Oncology, Institute of Pediatric Oncology and Hematology, Moscow, Russia; 49 Department of Haematology, Saint Antoine Hospital, Paris, France; 50 Neuroimmunology and MS Research, Neurology Clinic, University Hospital, Zurich, Switzerland; 51 Department of Clinical Immunology, National Referral Center for Autoimmune Diseases, Strasbourg University Hospital, Strasbourg, France; 52 Haematology Department, St. Vincent’s Health Network, Darlinghurst, NSW Australia; 53 Haematology Department, St. Vincent’s Health Network, Darlinghurst, NSW Australia; 54 Anthony Nolan Research Institute, London, UK; 55 Department of Brain Sciences, Imperial College London, London, UK; 56 Division of Clinical Immunology, Ribeirão Preto Medical School, University of São Paulo, Ribeirão Preto, Brazil; 57 Raisa Gorbacheva Memorial Research Institute for Pediatric Oncology, Hematology and Transplantation First State Pavlov Medical University of Saint Petersburg, Saint Petersburg, Russia 197022; 58 Haematology—BMT Centre, Fondazione IRCCS Ca’ Granda Ospedale Maggiore Policlinico, Università degli Studi di Milano, Milan, Italy; 59 Division of Hematology, Ribeirão Preto Medical School, University of São Paulo, Ribeirão Preto, Brazil; 60 Centre Hospitalier Universitaire de Poitiers, Poitiers, France; 61 University Hospital St. Marina, 9010 Varna, Bulgaria; 62 BMT Unit, Department of Hematology, IDIBAPS, Hospital Clinic, Institut Josep Carreras, Barcelona, Spain; 63 Department of Clinical Haematology, Manchester Royal Infirmary, Manchester, UK; 64 Bone Marrow Transplantation Unit, George Papanicolaou General Hospital, Thessaloniki, Greece; 65 Department of Hematology, Oncology and Internal Medicine, Medical University of Warsaw, Warsaw, Poland; 66 Department of Rheumatology, Leiden University Medical Centre, Leiden, Netherlands; 67 Division of Hematology and Hematopoietic Stem Cell Transplantation, Hematology and Stem Cell Transplantation Unit, Clinica FOSCAL, Bucaramanga, Colombia; 68 Divisie Kinderen, Cluster Immunologie, Reumatologie, Hematologie en Infectiologie, Wilhelmina Kinderziekenhuis, Utrecht, Netherlands; 69 Osp. G. Pini, Department of Rheumatology, Milan, Italy

**Keywords:** Peripheral neuropathies, Bone marrow transplantation

## Abstract

These updated EBMT guidelines review the clinical evidence, registry activity and mechanisms of action of haematopoietic stem cell transplantation (HSCT) in multiple sclerosis (MS) and other immune-mediated neurological diseases and provide recommendations for patient selection, transplant technique, follow-up and future development. The major focus is on autologous HSCT (aHSCT), used in MS for over two decades and currently the fastest growing indication for this treatment in Europe, with increasing evidence to support its use in highly active relapsing remitting MS failing to respond to disease modifying therapies. aHSCT may have a potential role in the treatment of the progressive forms of MS with a significant inflammatory component and other immune-mediated neurological diseases, including chronic inflammatory demyelinating polyneuropathy, neuromyelitis optica, myasthenia gravis and stiff person syndrome. Allogeneic HSCT should only be considered where potential risks are justified. Compared with other immunomodulatory treatments, HSCT is associated with greater short-term risks and requires close interspeciality collaboration between transplant physicians and neurologists with a special interest in these neurological conditions before, during and after treatment in accredited HSCT centres. Other experimental cell therapies are developmental for these diseases and patients should only be treated on clinical trials.

## Introduction

### Multiple sclerosis (MS)

MS is the most common chronic inflammatory demyelinating disease of the central nervous system (CNS) and the leading cause of non-traumatic neurological disability of young adults [1]. It affects ~2.3 million people worldwide with a prevalence of 1 in 700 adults [2]. Following diagnosis, patients rapidly fall out of employment, with recent data indicating that after 5 years only 25% of people are still working. As a result, MS has an economic impact disproportionate to its prevalence related to the high cost of disease modifying therapies (DMTs), the direct and indirect costs of relapses and associated costs of benefits and personal care [3].

MS is typically a biphasic disease. In the intial phase, the illness usually runs a relapsing remitting (RRMS) course [4] characterised by repeated episodes of inflammation within the CNS, often accompanied by Gadolinium (Gd) enhancing lesions on magnetic resonance imaging (MRI) and characterised pathologically by inflammatory infiltrates rich in T and B cells and macrophages [1]. The ensuing secondary progressive MS (SPMS) phase is characterised by slow accumulation of disability with a progressive decline in inflammation, and increasing axonal and neuronal loss [5]. Other clinical variants include primary progressive MS (PPMS) where patients experience disability progression from disease onset [4], and aggressive (or malignant) MS where the illness runs a fulminant course with rapid accumulation of significant disability [6]. The Expanded Disability Status Scale (EDSS) [7] is the most commonly used method of assessing disability progression in MS, whilst MRI is used to assess disease activity and atrophy.

Inflammatory forms of MS respond to immunomodulation with DMTs that aim to achieve a state of No Evidence of Disease Activity (NEDA), reflected by absence of clinical relapses, disability progression and MRI disease activity [8]. In the majority of patients with RRMS, the illness can be controlled by currently approved DMTs and various professional guidelines are available with recommendations for their sequential use based on baseline disease activity and response to treatment [9]. However, a significant proportion of patients continue to have clinical and/or MRI disease activity despite the use of DMTs [10]. Whilst more efficacious DMTs may lead in many but not in all patients to relatively high levels of disease control in the short term reflected by NEDA, these agents are expensive and have significant risks including infusion-associated reactions, secondary autoimmunity and infections including progressive multifocal leukoencephalopathy (PML). Unfortunately, the treatment options are very limited once the neurodegenerative phase of SPMS is established [11]. Equally, PPMS is very challenging to treat although some patients with clinical and MR scan activity may respond to immunomodulation [12].

There is increasing published evidence, including randomised controlled trials (RCTs), which convincingly demonstrates robust clinical efficacy of autologous HSCT (aHSCT) in patients with highly active MS, along with improved safety with markedly reduced levels of non-relapse mortality (NRM) risk, which supports its incorporation into standard MS treatment algorithms [13–21].

### Other neuroinflammatory diseases

Autoimmunity and neuroinflammation may affect the CNS and peripheral nervous systems (PNS) in a range of diseases including chronic inflammatory demyelinating polyneuropathy (CIDP), neuromyelitis optica (NMO), myasthenia gravis (MG), stiff person syndrome (SPS) and autoimmune encephalopathies [17]. There are also patients with systemic autoimmune diseases (ADs), where there is a significant neuroinflammatory component managed in neurology clinics. Whilst many patients respond well to standard treatment pathways, responses may be inadequate leading to the development of significant and potentially permanent disability consequent upon degenerative changes. In such settings aHSCT has been reported as a means of intensive immunomodulation [13, 18, 19, 21].

## Activity of HSCT in ADs: the EBMT Registry and the EBMT activity survey

The activity of HSCT and cell therapy in Europe is reflected by two complementary but different database analyses; the EBMT Registry, for which full EBMT membership mandates reporting of detailed data, and the broader EBMT activity survey, which captures annual HSCT activity, both from all EBMT members (full and associate) and other non-EBMT centres. Severe treatment-resistant ADs, predominantly MS, have been treated with both aHSCT and allogeneic HSCT (allo-HSCT) for over two decades and are currently the fastest growing indication group for HSCT in the annual EBMT activity survey [21–23].

**Fig. 1:**
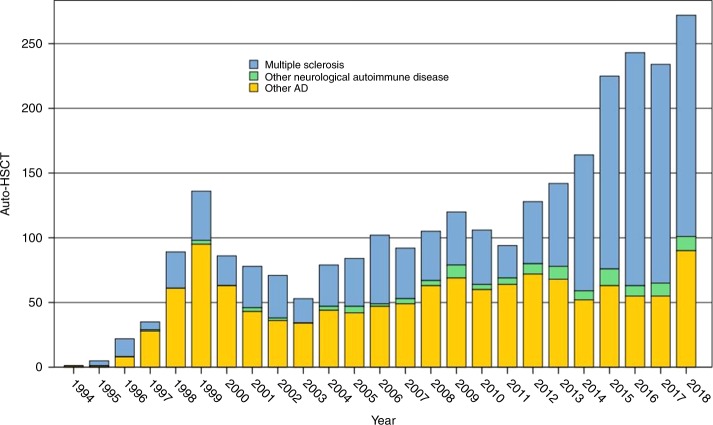
EBMT ADWP activity—autologous HSCT for MS, other immune-mediated neurological diseases and other autoimmune diseases by year, 1994–2018 (*N* = 2766)

**Fig. 2:**
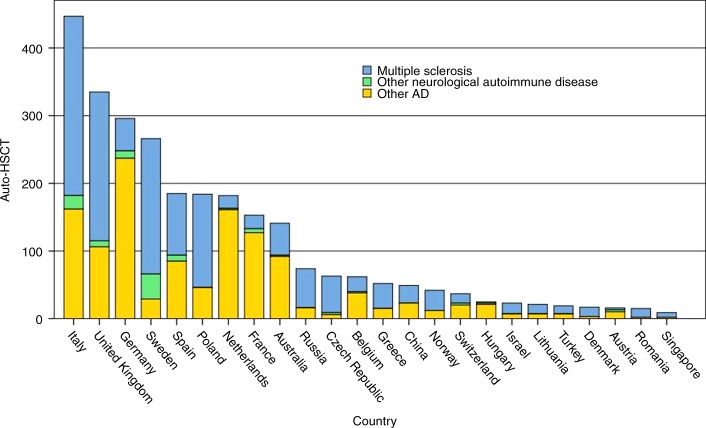
EBMT registry: overall national activity in autologous HSCT indicated for MS, other immune-mediated neurological diseases and other autoimmune diseases by country, 1994–2018 (*N* = 2766)

**Fig. 3:**
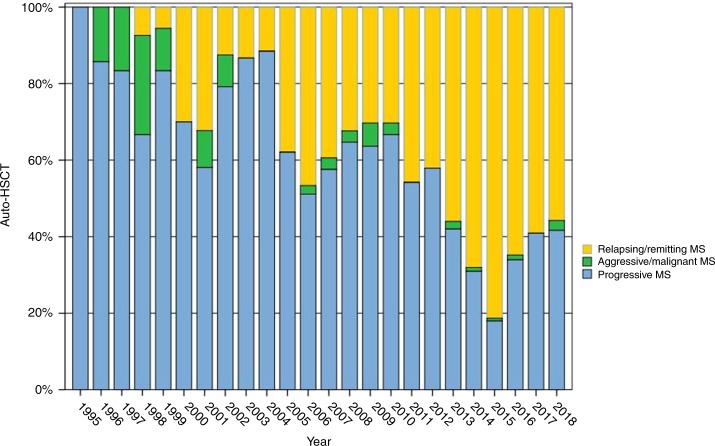
EBMT registry: relative activity according to reported multiple sclerosis type: RR-MS versus progressive MS (SPMS/PPMS) versus aggressive/malignant MS

The EBMT Registry is currently the largest database worldwide for HSCT with over half a million registrations, including over 3000 patients treated for autoimmune and inflammatory diseases. The current status of the EBMT Registry in relation to MS and other immune-related neurological diseases is summarised in Table 1 and Figs. 1–3, alongside the increasing activity in other ADs. There have been various degrees of uptake by national neurological and HSCT communities across EBMT, but overall the growing evidence base is reflected by a progressive increase in registrations, particularly in the last 5 years. Over time there has been a shift from SPMS to RRMS (Fig. 3). Paediatric patients (<18 years) undergoing aHSCT for MS are rare, with only 28 registrations to date.

**Table 1:** Summary of autologous HSCT for MS and other immune-mediated neurological diseases in the EBMT Registry, July 2019

	*N* (%)
**Multiple sclerosis**	**1446 (92.9)**
Malignant/aggressive	37 (2.7)
Progressive (primary or secondary)	617 (45.8)
Relapsing remitting	693 (51.4)
Missing (*n* *=* 99, 6.8%)	
**Other neurological disease**	**105** (**7.1)**
Chronic inflammatory demyelinating polyneuropathy	54 (3.5)
Neuromyelitis optica	17 (1.1)
Myasthenia gravis	9 (0.6)
Encephalitis	5 (0.3)
Stiff person syndrome	4 (0.3)
Other neurological diseases	21 (1.3)

NRM, an unfamiliar concept to most neurologists, is used interchangeably in these guidelines with the closely-related treatment-related mortality (TRM) parameter, and is an important consideration for HSCT in immune-mediated neurological diseases, which may be severely disabling but only rarely immediately life-threatening. In MS, NRM (and TRM) have significantly improved significantly in EBMT registry data [21], with recently reported levels of 0.2%, similar to levels derived from meta-analysis of published studies [16], and this may be attributed to greater experience, patient selection, transplant technique and accreditation [13–21].

It is not possible to provide meaningful estimates of the activity trends and NRM risks of aHSCT in the rarer immune-mediated neurological diseases given small numbers, heterogeneity and varying degrees of disability and co-morbidity. The published literature includes some outcomes and may be open to selection bias. These rare indications are the subject of ongoing EBMT registry-based analyses. Likewise, the numbers of patients who have received allo-HSCT for neurological ADs are low [21], even in a recent EBMT analysis of allo-HSCT [24].

## EBMT guidelines and recommendations

Multi-disciplinary guidelines across a wide range of ADs were published by the EBMT ADWP in 1997 and 2012 to cover general principles of patient selection, stem cell collection, graft manipulation, conditioning regimens, supportive care and follow-up [25, 26]. These included guidelines for MS and other immune-mediated neurological diseases, but, given the increase in evidence, updates are now warranted. The EBMT has recently published a broad update of all malignant and non-malignant indications for HSCT, which covers the main adult and paediatric ADs but provides limited detail [27].

The aim of these updated guidelines is to provide a more detailed and comprehensive review of the evidence, registry data and mechanisms of action and to provide specific recommendations for patient selection, treatment procedures, follow-up and future development of HSCT in patients with MS and other immune-mediated neurological diseases. As previously, the guideline authorship group includes clinicians from relevant professional groups active in the ADWP, including nursing, statistical and data management representation, all with experience in HSCT for neurological ADs. The principal target audience is transplant physicians, nurses and their teams as well as neurologists working with transplant teams, or considering referral of patients. The guideline is not primarily targetted at patients, families and non-specialist health professional carers, although it supplements recently published information from the EBMT [28]. Evidence was sourced from PubMed searches of original observations and key reviews and, where relevant, recent EBMT congress presentations, with a view to updating the previous EBMT 2012 guidelines [26]. As per other EBMT guidelines and recommendations [26, 27], evidence for indications is systematically classified in four categories where HSCT should be considered (S/CO/D/GNR—see Table 2 and related footnotes). Strength of the evidence supporting the assignment of a particular category is graded (levels I, II and III) based on consideration of health benefits, side effects and risks and balanced against the non-HSCT options. Each recommendation provides potential for auditing clinical practice. The guideline also considers the resource implications and other issues relevant to implementation of HSCT in this area. Other than EBMT support there is no funding body supporting these guidelines, commercial or otherwise, and conflicts within the authorship are disclosed. The EBMT ADWP plan future updates according to developments in evidence base and clinical practice.

**Table 2:** Summary of recommendations for HSCT and cellular therapy in multiple sclerosis and other immune-mediated neurological diseases

	Autologous HSCT	MSD Allo HSCT	MUD Allo HSCT	MMAD Allo HSCT	Cellular therapy
Highly active relapsing remitting MS failing DMTs	S/I	D/III	GNR/III	GNR/III	D/III
Progressive MS with active inflammatory component	CO/II	D/III	GNR/III	GNR/III	D/III
Aggressive^a^ (malignant) MS not previously treated with a full course of DMT	CO/II	D/III	GNR/III	GNR/III	D/III
Progressive MS without active inflammatory component	GNR/III	GNR/III	GNR/III	GNR/III	D/III
Paediatric MS	CO/II	GNR/III	GNR/III	GNR/III	D/III
CIPD	CO/II	GNR/III	GNR/III	GNR/III	D/III
NMO	CO/II	D/III	D/III	D/III	D/III
MG	CO/II	GNR/III	GNR/III	GNR/III	D/III
SPS	CO/II	GNR/III	GNR/III	GNR/III	D/III
Systemic ADs e.g. SLE, vasculitis, Behcet’s disease, Sjogren’s syndrome, refractory coeliac disease with neurological manifestations	CO/II	GNR/III	GNR/III	GNR/III	D/III

As updated by Duarte et al. [27], EBMT indications are classified in four categories, listed below, to describe the settings where these types of transplants ought to be performed. The strength of the evidence supporting the assignment of a particular category is graded in three levels:

Grade I: 181 Evidence from at least one well-executed randomised trial

Grade II: Evidence from at least one well-designed clinical trial without randomisation; cohort or case-controlled analytic studies (preferably from more than one centre); multiple time-series studies; or dramatic results from uncontrolled experiments

Grade III: Evidence from opinions of respected authorities based on clinical experience, descriptive studies, or reports from expert committees

Standard of care (S): Indications categorised as S are reasonably well defined and results compare favourably (or are superior) to those of non-transplant treatment approaches. Obviously, defining an indication as the standard of care does not mean an HSCT is necessarily the optimal therapy for a given patient in all clinical circumstances. “Standard of care” transplants may be performed in a specialist centre with experience in HSCT and an appropriate infrastructure as defined by the JACIE guidelines

Clinical option (CO): The CO category applies to indications for which the results of small patient cohorts show efficacy and acceptable toxicity of the HSCT procedure, but confirmatory randomised studies are missing, often as a result of low patient numbers. The broad range of available transplant techniques combined with the variation of patient factors such as age and co-morbidity makes interpretation of these data difficult. Our current interpretation of existing data for indications placed in this category supports that HSCT is a valuable option for individual patients after careful discussions of risks and benefits with the patient but that for groups of patients the value of HSCT needs further evaluation. Transplants for indications under this heading should be performed in a specialist centre with major experience in HSCT with an appropriate infrastructure as defined by JACIE guidelines

Developmental (D): Indications have been classified as D when the experience is limited, and additional research is needed to define the role of HSCT. These transplants should be done within the framework of a clinical protocol, normally undertaken by transplant units with acknowledged expertise in the management of that particular disease or that type of HSCT. Protocols for D transplants will have been approved by local research ethics committees and must comply with current international standards. Rare indications where formal clinical trials are not possible should be performed within the framework of a structured registry analysis, ideally an EBMT non-interventional/observational study. Centres performing transplants under this category should meet JACIE standards

Generally not recommended (GNR): The GNR category comprises a variety of clinical scenarios in which the use of HSCT cannot be recommended to provide a clinical benefit to the patient, including early disease stages when results of conventional treatment do not normally justify the additional risk of a HSCT, very advanced forms of a disease in which the chance of success is so small that does not justify the risks for patient and donor, and indications in which the transplant modality may not be adequate for the characteristics of the disease. A categorisation as GNR does not exclude that centres with particular expertise on a certain disease can investigate HSCT in these situations. Therefore, there is some overlap between GNR and D categories, and further research might be warranted within prospective clinical studies for some of these indications

^a^Aggressive MS as per Menon et al. [6]

## Clinical evidence for aHSCT in MS and immune mediated neurological diseases

### Multiple sclerosis (MS)

Although the first patients to be treated with aHSCT for MS were in 1995 [29, 30], there is now growing evidence from large registry studies and two prospective comparative trials to support the efficacy of aHSCT in patients with highly active MS, with long-term clinical and MRI remissions observed in a majority of patients with acceptable safety. These include (1) a small phase II RCT that, despite some methodological limitations, demonstrated the superiority of aHSCT with the ‘BEAM-ATG’ intermediate intensity conditioning regimen in suppressing MRI activity and clinical relapses compared with mitoxantrone [31]; (2) single arm prospective studies demonstrating aHSCT with cyclophosphamide-ATG (‘Cy-ATG’), ‘BEAM-ATG’, or high-intensity (‘BuCy-ATG’) conditioning regimens induced sustained clinical remissions and suppression of MRI activity in patients with active MS [32–35].

Similar outcomes were reflected in other large retrospective series [15, 36–38]. Long-term outcomes have been analysed in a large cohort of patients treated before 2006, which included a mixture of RRMS, SPMS and PPMS [15]. Systematic analyses of NEDA rates following aHSCT support durable clinical remission in a high proportion of patients with RRMS, suggesting that potential benefit could exceed that seen after approved DMTs including those considered to be highly efficacious [39, 40].

The evidence-base has been significantly boosted by the recent publication of interim results of the first large RCT phase III study, MIST, comparing aHSCT using a non-myeloablative regimen (Cy-ATG) versus FDA approved DMTs with no deaths or serious toxicity in the HSCT group [41]. Moreover, 30 patients who were originally randomised into the DMT arm were crossed over to the transplant arm after reaching the primary endpoint of the trial, with significant fall in EDSS after receiving aHSCT [42].

The interim results of MIST provide evidence that aHSCT is safe and has superior efficacy compared with many currently available DMTs, although, for historical reasons, MIST did not include alemtuzumab, ocrelizumab or cladribine in the control arm. Therefore, there remains a need for comparative studies that randomise patients to aHSCT versus these agents [43–45]. Even so, it would appear that aHSCT still offers clear advantages with NEDA rates of 66–93% compared with alemtuzumab, natalizumab or ocrelizumab. The area needs to be systematically resolved via prospective RCTs (see section Clinical trials of aHSCT in MS).

### Patient selection for aHSCT in MS

Undoubtedly aHSCT is more intensive and has greater short-term toxicities than any DMT. It is used in MS primarily as an anti-inflammatory and immunomodulatory treatment, which makes the presence of significant clinical and MRI evidence of an active inflammatory component, along with fitness to tolerate it, a pre-requisite. Younger patients, shorter duration of disease, lower EDSS scores, active inflammatory disease, and absence of other co-morbidity have been associated with favourable outcomes [15, 16, 18–20, 27, 39, 40, 46–48]. Any decision to proceed must assess the balance of benefits and risks particularly in terms of reversibility or stabilisation of disability and other neurological features.

#### Highly active relapsing remitting MS failing DMT

In line with MIST and other studies, patients with highly active RRMS failing at least one line of DMT may be considered for aHSCT, with treatment failure defined by the documented occurrence of at least two clinical relapses or one clinical relapse and the presence of MRI activity at an independent time point in the previous 12 months [16, 18, 20, 41, 42].

#### ’Aggressive’ MS

About 4–14% of MS patients have ‘aggressive’ disease and experience an accelerated (3–4 times faster) disease course [6, 49]. Various terminologies have been used to describe this ‘aggressive’ phenotype, including ‘malignant’, ‘fulminant’ and ‘Marburg variant’. The ‘therapeutic window’ in a patient with ‘aggressive’ MS is significantly shorter and, in this relatively rare context, aHSCT is highly effective at inducing prolonged clinical remissions [50–52]. Thus, deteriorating patients with ‘aggressive’ disease at risk of irreversible disability should be rapidly considered for aHSCT, even if a full course of DMT has not been completed to formally establish treatment failure [20, 26, 50].

#### Progressive MS with active inflammatory component

Registry studies and other cohort analyses have repeatedly shown that aHSCT is more efficacious in patients with RRMS than SPMS or PPMS [13–16, 18, 20, 26, 27]. Even so, several reports support the association of Gd-enhancement with favourable outcomes [15, 16, 31, 53]. More recent data from the siponimod trial [11] support a role for ongoing inflammation in the chronic progressive phase of MS and aHSCT may therefore be justified at this stage provided that disease activity has been documented.

In PPMS, registry-based studies have supported very limited benefit with aHSCT, if at all, and therefore recommendations have previously discouraged its use [26]. However, some studies have suggested that immunomodulation may provide benefit [54, 55]. More recently, treatment with ocrelizumab has been associated with lower rate of clinical and MRI progression [12]. Given the poor prognosis, the support from registry data [15, 16] and the limited treatment options, very occasional patients with high levels of persistent inflammatory activity with rapidly accumulating disability may be considered. Prospective studies are warranted to explore the potential of aHSCT in PPMS.

#### Paediatric MS

MS is a rare disease in children, but its consequences are particularly severe as disability may be life-long [56]. In a cohort of 21 patients under 18 years, aHSCT was well tolerated and associated with improvements of EDSS scores in 81% of patients with progression free survival (PFS) of 100% at 3–5 years, hence potentially more efficacious in children than in adults [17]. Given a greater potential for late effects, a reasonable approach is to try other less toxic treatments first, e.g. interferon or fingolimod [57], and reserve aHSCT for patients with breakthrough inflammation.

## Recommendations

- aHSCT should be offered to patients with RRMS with high clinical and MRI inflammatory disease activity (at least 2 clinical relapses, or one clinical relapse with Gd-enhancing or new T2 MRI lesions at a separate time point, in the previous 12 months) despite the use of one or more lines of approved DMTs. Evidence best supports treatment in patients who are able to ambulate independently (EDSS 5.5 or less), who are younger than 45 years and have disease duration less than 10 years (level S/I).
- Patients with ‘aggressive’ MS, who develop severe disability in the previous 12 months, are suitable candidates for aHSCT. Given the potential for irreversible disability, such patients may be considered even before failing a full course of DMT (level CO/II).
- Patients with SPMS should be considered for aHSCT, preferably in a prospective clinical trial, only when inflammatory activity is still evident (clinical relapses and Gd-enhancing or new T2 MRI lesions) with documented disability progression in the previous 12 months (level CO/II).
- Patients with PPMS should be considered for aHSCT, preferably in a prospective clinical trial, only when inflammatory activity is evident (Gd-enhancing and new T2 MRI lesions) with documented evident disability progression in the previous 12 months (level CO/II).
- Paediatric patients with MS who have breakthrough inflammatory disease with less toxic treatments may be considered for aHSCT (level CO/II).

### aHSCT in other immune-mediated neurological diseases

#### Chronic inflammatory demyelinating polyneuropathy (CIDP)

CIDP is an immune-mediated disease targeting peripheral nerves. To prevent disability, immunosuppressive treatments should be initiated before irreversible axonal damage has occurred. There is limited experience with aHSCT in CIDP with a total of 20 patients reported (four received BEAM-based, and the remainder cyclophosphamide-based protocols) of whom 90% improved, and 35% experienced further relapses [58–62]. Recently, a large single centre experience was reported with high levels of response [63].

#### Myasthenia gravis (MG)

MG, an immune-mediated disease targeting the neuromuscular junction, has been treated with aHSCT, with ten patients described in the literature. Seven were treated at a single centre with high-intensity conditioning regimens containing total body irradiation (TBI) or busulphan, with good tolerance and durable remission in all patients after a median follow-up of 40 months [64]. Similar outcomes in three further patients using cyclophosphamide-based conditioning were reported [65–67].

#### Stiff person syndrome (SPS)

SPS is a rare immune-mediated neurological disorder characterised by muscle rigidity, spasms, brain stem hyperexcitability and high glutamic acid decarboxylase-specific antibodies. aHSCT has successfully been used to treat limited numbers of SPS patients [68, 69]. Most patients respond to aHSCT, although responses are variable and may depend on the variant and duration of SPS.

#### Neuromyelitis optica (NMO)

NMO is an inflammatory autoimmune disorder of the CNS, characterised by pathogenic anti-aquaporin four antibodies (AQP-4Ab) and a generally worse prognosis than MS. The EBMT summarised 16 patients with refractory NMO treated with aHSCT (treated mainly with the ‘BEAM-ATG’ regimen); three cases remained progression- and treatment-free, whilst anti-AQP-4Ab antibodies persisted in 13 patients who required further treatments for relapses or disability progression [70]. Other data come from two case reports and a Chinese study in 21 patients with so-called opticospinal MS [71–73]. A recent case report showed a sustained clinical, radiological and immunopathological NMO remission with rituximab treatment prior to aHSCT [74]. Recent data from Northwestern University support favourable clinical outcomes of aHSCT with the Cy-ATG regimen combined with rituximab, with clearance of anti-AQP-4Ab [75].

#### Other immune-mediated neurological diseases

Autoimmune encephalitis and other rare neurological diseases treated with aHSCT feature in the EBMT registry (see Table 2), but published reports are limited.

#### Systemic ADs with neurological manifestations

In addition to autoimmune neurologic diseases, rheumatic diseases with CNS or PNS involvement and insufficient response to conventional immunusuppressive or biologic therapies represent a growing indication for aHSCT. Where there is a significant or predominant neurological component, they may be managed in neurology clinics.

In a recently published study presenting the outcomes of aHSCT in 30 patients with SLE, ten patients suffered from neuropsychiatric manifestations, responding to aHSCT with cyclophosphamide, rabbit ATG and rituximab [76]. Similar results are obtained in smaller case series, which are summarised in a retrospective EBMT survey [77].

Systemic vasculitis may have neurological manifestations. Published literature on aHSCT for refractory BD with severe CNS involvement includes two patients from a retrospective data analysis from the EBMT registry [78] and smaller series including one case undergoing first autologous followed by allogeneic HSCT [79]. All patients achieved complete remission, but one patient relapsed 2 years after HSCT. Data on Granulomatosis with Polyangiitis (formerly Wegener’s granulomatosis) with CNS involvement is limited to a single case reported to the EBMT registry, which achieved a complete response following conditioning with Cy-ATG and CD34-selected aHSCT [80]. Sjogren’s syndrome, polymyositis-dermatomyositis and refractory coeliac disease (RCD) with neuromuscular manifestations have also been treated with aHSCT with favourable responses reported [61, 81–85].

Recommendations

- Patients with refractory CIDP, MG, NMO, SPS and systemic AD with neurological manifestations may be considered for aHSCT (level CO/II).

## aHSCT procedure

### General principles

#### Centre experience and accreditation

aHSCT is an intensive procedure with a level of immediate transplant-related risks and other toxicities. Registry studies support a positive impact of JACIE accreditation [86] on PFS, whilst the centre experience in ADs resulted in a statistically significant improvement of TRM/NRM, PFS and overall survival [15, 21]. Such improvement is likely related to progressively improved patient selection, a dedicated pattern of care and the full integration between the HSCT and disease specialists. Experience is important as conditioning regimens used in aHSCT in ADs induce more profound immunosuppression than in haemato-oncological indications due to ATG, with a higher incidence of acute reactions, viral reactivations and infections. In addition, administration of DMTs before aHSCT may have an impact on the graft characteristics and immune reconstitution and further studies are required. There is a need for an extended competency and package of care for neurological patients, including specific pre-transplant work-up with attention to cardio-respiratory function, specific neurological supportive care measures, prolonged infective monitoring after the procedure, consideration of physiotherapy/rehabilitation [26]. Centre experience and accreditation may improve patient care and outcomes via implementation of specific staff training, procedures and audit in the institutional quality management system [21, 86].

Recommendations

- aHSCT should be delivered in transplant units that provide high quality care and are accredited by JACIE or equivalent organisations (level II).
- Units should be experienced with close collaboration between HSCT and neurology specialists throughout the patient journey including medium- and long-term follow up (level II).

#### Multidisciplinary teams (MDTs) and patient consent

Any decision to proceed must assess the balance of benefits and risks particularly in terms of reversibility or stabilisation of disability and other neurological features. Decision-making requires critical multidisciplinary input from neurology and haematology specialities and may also involve other core members, such as nursing and professions allied to medicine (PAMs).

Informed consent should be obtained for all phases of the transplant procedure, A frank discussion about potential risks, including TRM risk, transient worsening of function and other early and late transplant-related toxicities is an essential part of the consent process. The discussion should also include the risk-benefit of alternative treatments, including DMTs. Patients with childbearing potential should be counselled appropriately as temporary or permanent ovarian/testicular failure and infertility following aHSCT are known risks [87, 88]. Fertility preservation strategies should be discussed. All patients should be invited to provide separate consent for submission of their anonymised/pseudonymised personal data to the EBMT, or equivalent, registry in accordance with relevant data protection and other regulations.

### Transplant technique

**Fig. 4:**
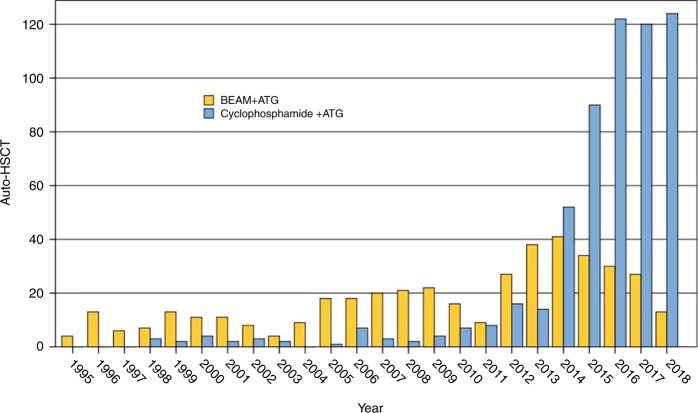
Trends in transplant conditioning used for autologous HSCT in Multiple Sclerosis: BEAM-ATG versus Cy-ATG (EBMT Registry 1995–2018)

A variety of transplant techniques have been used, both in mobilisation and conditioning (Table 3). In accordance with previous EBMT guidelines [21, 26], two ‘intermediate-intensity’ conditioning regimens have been used most commonly in MS: BEAM-ATG and cyclophosphamide 200 mg/kg + ATG (Fig. 4). Data on transplant technique for aHSCT in other immune-mediated neurological disorders outside MS is limited and heterogeneous.

**Table 3:** Categorisation of conditioning regimens used for autologous HSCT, with examples used in MS and other immune-mediated neurological diseases [20, 21, 26]

Intensity	Examples of conditioning regimens
High	Total body irradiation (TBI), cyclophosphamide and ATG
	Busulfan, cyclophosphamide and ATG (BuCyATG)
Intermediate (myeloablative)	Carmustine (BiCNU) 300 mg/m^2^, etoposide 800 mg/m^2^, cytarabine arabinoside 800 mg/m^2^ and melphalan 140 mg/m^2^ (BEAM, with total doses of chemotherapy provided) and ATG (‘BEAM-ATG’)
Intermediate (lymphoablative/non-myeloablative)	Cyclophosphamide 200 mg/Kg and rabbit ATG (Cy-ATG)
Low	Chemotherapy only^a^ regimens e.g. single agent cyclophosphamide 100 mg/kg for mobilisation and repeated 100 mg/kg for conditioning (without rituximab) [96, 97]

Please note doses are examples and the authors do not take responsibility for drug and doses administered, which lies with individual authorised prescribers in HSCT units. Doses and types of ATG vary between published regimens

^a^Addition of serotherapy (i.e. antibody therapy) to chemotherapy renders the regimen ‘intermediate-intensity’)

#### Pre-transplant ‘wash-out’

Prior to mobilisation, DMTs and other immunomodulatory drugs should be discontinued as early as possible, which may help minimise risks and inhibitory effects on successful mobilisation. ‘Wash-out’ periods, commonly used in neurological practice for switching between DMTs, aim to reduce the risks of PML and other infections [89]. There is no consensus to support duration of wash-out periods. The following ‘wash-outs’ are examples; at least 6 weeks for dimethyl fumarate, fingolimod and natalizumab, and 6 months for alemtuzumab, ocrelizumab and cladribine given the more profound lymphopenia and risk of infection. Accelerated elimination should be considered in patients on teriflunomide (https://www.aubagiohcp.com/content/pdf/drug_elimination_guide.pdf). No wash-out is necessary for interferon and glatiramer acetate. There have been no reports of PML following aHSCT in current EBMT registry data, but CSF JCV-PCR should be done on patients transitioning from natalizumab if they have high JCV antibody Index. Steroid pulses may be used to reduce the risk of relapses during the wash-out period.

#### Peripheral blood stem cell [PBSC] mobilisation and leukapheresis

Most patients treated for AD have received priming doses of cyclophosphamide of 2–4.5 g/m^2^ with uromixetan (Mesna) and/or cautious hyperhydration followed by G-CSF 5–10 μg/kg prior to leukapheresis [26, 29–38, 41]. Administration of G-CSF alone may induce disease flare, but its combined administration with ‘priming’ chemotherapy usually prevents flares, reduces T-cell numbers in the graft and improves PBSC yields [90]. There are no data in terms of efficacy, but cyclophosphamide at a dose of 2 g/m^2^ is likely to be safer than higher doses but potentially less effective in terms of both mobilisation potential and disease control. The procedure can usually be carried out as an outpatient regimen, but in disabled patients hospital admission may be considered. The need for repeat harvest appears to be rare, with little data to support the need for off-licence use of plerixafor.

In line with EBMT recommendations, the minimum dose of CD34^+^ cells for re-infusion is 2.0 × 10^6^/kg, although other generic recommendations have proposed 4−5 × 10^6^/kg as the optimal dose [91, 92]. Considering that MS and neurological disorders are non-malignant indications, it would be pragmatic to aim for 5 × 10^6^/kg as an optimal target before freezing, with 2.0 × 10^6^/kg as a minimum safety threshold. Doses higher than 8 × 10^6^/kg are unlikely to improve the rate of engraftment and have a theoretical risk of increased T cell contamination of the graft.

Neurological patients undergoing mobilisation are at risk of febrile neutropenia during mobilisation, and, if fever occurs, there may be a related transient worsening of neurological function, referred to as the Uhthoff phenomenon [93]. Oral antibiotic prophylaxis should be considered with a rapid pathway for hospital readmission and treatment of fever including use of steroids. Where disability precludes rapid readmission, patients can be hospitalised for the mobilisation phase.

#### Conditioning regimens

Previous EBMT ADWP recommendations recommended the use of ‘intermediate intensity’ regimens namely cyclophosphamide 200 mg/kg with T-cell depleting serotherapy (most commonly ATG) as a generic regimen across ADs, and, for MS, ‘BEAM-ATG’, was specifically recommended (Table 3) [26]. The use of ‘high-intensity’ regimens including TBI or busulfan was not recommended on grounds of short and long-term toxicity, whilst the ‘low-intensity’ regimens were considered to be less efficacious [21, 26]. Higher intensity regimens, such as the ‘BuCy-ATG’ regimen, are efficacious but have been associated with potentially serious side effects, including veno-occlusive disease [34]. TBI, with its greater short and long-term risks, including infections, secondary malignancies, NRM and EDSS progression possibly due to radiation neurotoxicity, is now rarely used, if at all, and was reported as ineffective in advanced MS [94]. Regimens of a lower intensity such as cyclophosphamide 120 mg/kg with ATG seem to be associated with an increased rate of relapse [95]. There is experience in Mexico of a low-intensity regimen where cyclophosphamide at 100 mg/kg has been used prior to re-infusion with unfrozen PBSC, with and without post-transplant rituximab. However, long-term outcome data are limited [96, 97].

Since the publication of the EBMT 2012 guidelines [26], there has been an increase in the use of Cy-ATG regimen in MS whilst BEAM-ATG usage has also been maintained (Fig. 4). At present, there is no comparative data as to the relative efficacy and safety of these two most commonly used intermediate-intensity conditioning regimens. Therefore, EBMT guidelines advocate using either of these two regimens for MS. The question of relative safety and efficacy between these two intermediate treatment regimens may be resolved through an ongoing EBMT registry analysis.

With respect to T-cell depleting serotherapy, the majority of MS patients have been treated with rabbit ATG (rATG) from various sources (Thymoglobulin/Sanofi-Genzyme and Grafalon/Neovii). Despite potential immunomodulatory advantages in non-transplant settings [98], the use of horse-ATG (hATG) has been limited compared with rATG and associated with a greater level of toxicity in one early study running from 2001–2006 [99]. However, in a more recent study the safety of a specific type of hATG (ATGAM, Pfizer) was assured with outcomes comparable to recent data using rATG [100]. The choice of type and dose of rATG depend on availability and centre preference, but in the published literature has been most commonly polyclonal rATG of Thymoglobulin type given in dose range of 5–7.5 mg/kg. Higher serum levels and type of ATG have been linked with infection and other outcomes in allogeneic HSCT [101–103] and non-transplant aplastic anaemia [104] settings, but this has not been systematically investigated in relation to aHSCT for ADs. Other forms of serotherapy, such as alemtuzumab, have been used, although data suggest a higher rate of complications including secondary autoimmunity [33]. Given the heterogeneity of types of ATG and other serotherapy, further evaluation of their use in conditioning regimens is urgently warranted.

Although HSCT units are likely to be experienced in the administration of ATG, it requires special attention given the potential for severe allergic-type reactions. These risks can be minimised with pre-medication consisting of antihistamines, paracetamol and steroids along with consideration of graduated dosing regimens and slow infusion rates. Varying doses of methylprednisolone (up to 1000mg [41]) have been used as pre-medication, but a minimum of methylprednisolone 2 mg/kg intravenously is recommended with a sufficient time interval (e.g. 30–60 min) before the start of the ATG infusion. As there is ongoing risk of ATG-related fever and other reactions after the infusion a tapering dose of oral or intravenous steroid is often used routinely, with breakthrough febrile or other episodes treated with additional pulses of intravenous methylprednisolone (e.g. 250 mg) whilst ensuring that infection is fully covered.

#### CD34^+^ selection and other graft manipulation

The question of graft manipulation is unclear and is confounded with inevitable but unquantifiable degree of in vivo depletion of T cells and other immune effector cells when ATG is included in the conditioning regimen. In MS, both unmanipulated and manipulated autologous grafts have been used. CD34^+^ selection has featured in some clinical trials, including in combination with the higher intensity BuCy-ATG regimen. Whether this contributes to the reported benefits and toxicity is unclear. An EBMT retrospective analysis failed to show benefit of graft manipulation in MS [105], and use in most other ADs [26]. Moreover, CD34^+^ selection may be associated with excess infection and the selection procedure adds significantly to the costs and logistics of aHSCT. In the absence of firm evidence of benefit, the recommendation is that CD34^+^ selection or other graft manipulation is not used outside a clinical trial setting in MS and other neurological diseases.

#### Supportive care, nursing and rehabilitation aspects

Most patients have nursing and supportive care (including transfusion) requirements common to patients undergoing aHSCT for other indications. The main difference in patients is the degree of baseline disability. In addition, the administration of conditioning chemotherapy and ATG with high-dose steroids and hyperhydration in most regimens requires close inpatient observations, including fluid and electrolyte balance. Twice-daily weighing is recommended. As some neurology patients are prone to seizures, some units incorporate prophylaxis against seizures during conditioning. The risk of potential physical and psychological side effects of high-dose steroids should be highlighted to both patients and nursing staff.

Urinary bladder dysfunction is common in MS, and residual volumes of urine represent not only a risk of infection, but also a risk of retaining cyclophosphamide metabolite, acrolein, which may cause haemorrhagic cystitis. All patients should be assessed for residual volume with ultrasound and, if necessary, a urinary catheter should be in situ for the period of cyclosphophamide administration. This should be accompanied by uromixetan (Mesna) as per departmental standard operating procedures. Patients with long-term indwelling catheters should be managed appropriately, with vigilance for the higher level of infection risk.

Occurrence of fever may affect the physical and mental state of the patient, and increase nursing needs to a greater degree in MS than in most other febrile transplant patients. Causes include ATG reactions, sepsis, urinary infections and viral reactivations. Fever of any type may temporarily compromise neurological function, referred to as the Uhthoff phenomenon [93], and sustained fever during the transplant period have been reported to affect long term efficacy [33]. Fever should be pro-actively managed appropriate to the clinical picture to induce rapid defervescence.

Vitamin D may have an impact on health and immune responses in MS and HSCT, and, given that patients are hospitalised during HSCT, routine supplementation should be considered [106].

Assessment and planning for rehabilitation should be performed prior to the transplant, for both the inevitable deconditioning effect of the aHSCT procedure and specific to neurological function of the patient. This area is currently the subject of a detailed EBMT ADWP review and guidance.

Recommendations

- All patients should be discussed within a MDT (level III).
- Informed written consent, including discussions regarding alternative therapeutic options, should be obtained in accordance with national and local regulatory and legal requirements (level III).
- Cyclophosphamide 2 g/m^2^ and G-CSF 5–10 μg/kg are recommended for mobilisation as they are likely to be sufficient for successful mobilisation, prevent flare and be potentially safer than higher doses (level II).
- For leukapheresis, an optimal target CD34^+^ cell dose is 5 × 10^6^/kg before freezing, with 2 × 10^6^/kg as a minimum safety threshold (level II).
- For conditioning, the use of ‘intermediate-intensity’ regimens namely cyclophosphamide 200 mg/kg + ATG or ‘BEAM-ATG’ is recommended (level II).
- The use of ‘high-intensity’ regimens, including TBI or busulfan, should be restricted to study protocols in highly selected patients (level II).
- De-escalated regimens may be less efficacious, but the balance of benefits and risks of such regimens should be established with clinical trials (level II).
- In the absence of high quality data in other immune-mediated neurological diseases, aHSCT technique should reflect the practice in MS depending on the experience of the transplant unit i.e. use of the EBMT recommended ‘generic’ regimen of cyclophosphamide 200 mg/kg + ATG or BEAM-ATG (choice depending on the experience of the transplant unit) with the addition of B-cell depleting monoclonal antibodies (such as rituximab) when the disease origin includes a relevant antibody-mediated component (level II).
- In the absence of firm evidence of benefit, CD34^ +^ selection or other graft manipulation should not be used outside a clinical trial setting (level II).
- Teams should be trained and competent with management of complications of the transplant regimen used in MS and other immune-mediated neurological diseases, including administration of and reactions to ATG and prevention and prompt management of fever in this context (level III).
- Given the deconditioning effect of the aHSCT procedure combined with neurological disability highlight rehabilitation requirements should be assessed before and during the transplant admission and in place at the time of discharge (level III).

## Early and late post-transplant follow up

aHSCT may be associated with both early and late complications or late effects [26, 107].

### Post-discharge monitoring and early post-transplant complications

The use of aHSCT in neurological disorders has key differences compared to other common indications, notably related to the neurological condition themselves and degree of immunosuppression [108]. Post-discharge monitoring is predominantly focused on infection in the first months after aHSCT with prophylaxis as per centre protocols akin to allo-HSCT recommended. Generally oral prophylaxis should cover fungal infections (with an azole) for 3 months and herpes virus (with aciclovir) and pneumocystis infection for a minimum of 6 months post-aHSCT, with many units extending to 12 months. Viral reactivation is important so PCR-based EBV/CMV monitoring is mandated during first 100 days. CMV re-activation occurs at a greater rate and cases of CMV infection have been reported. EBV reactivation usually resolves spontaneously, but may need treatment with rituximab and may be associated with neurological events and de-novo paraproteinemia [109]. Immune monitoring of T- and B-cell subsets and immunoglobulin levels/electropheresis is recommended on a 3-monthly basis in the first year and then annually in order to guide infection prophylaxis and detect paraproteinaemia [110].

Transient alopecia and amenorrhoea are common adverse effects, but menstrual function may recover especially in younger patients (<30 years of age) [88]. Haemorrhagic complications (e.g. gastrointestinal bleeding, hemorrhagic cystitis) have been reported.

### Late effects/long-term complications

International guidelines and recommendations cover screening and management of ‘late effects’ following HSCT [107, 111]. Late effects following aHSCT may result from the transplant regimen and altered post-transplant immune reconstitution, but may also be driven by pre-treatment of the underlying neurological disease. Since 2012 ‘late effects’ follow-up has been highlighted in the EBMT ADWP guidelines [26], but limited data is available on the frequency and nature of late effects following aHSCT above what would be expected in the general population, and also what would be expected in the MS population treated with DMTs [44, 112]. Impact on gonadal function and fertility should have been covered counselling related to the informed consent process, but should be revisited in routine follow-up of late effects [87, 88]. Other recognised late effects include secondary autoimmunity (up to 10%) either de novo or within the spectrum of the original AD [41, 113–115], endocrinopathy [33, 41] and late cancers [15, 35]. Concurrent AD is not infrequent and an appropriate screening (e.g. thyroid function) at baseline is mandatory. Although data are limited, the risk of PML appears low, with no current reports post-aHSCT, including in over 1400 patients treated for MS in the EBMT registry despite the frequent use of DMTs prior to transplantation (Table 1). Late effects are the subject of ongoing EBMT retrospective studies, but in the meantime, it is important that systematic screening is undertaken in accordance with current recommendations for late effects [26, 107].

### Post-transplant vaccinations

Vaccination post-HSCT is a balance between reducing the risk of infection but comes with a theoretical risk of triggering immune events, which is a concern in the setting of ADs [116, 117]. Vaccination practice varies [118], but in general, only vaccinations with live attenuated viruses are considered to pose a higher risk of inducing a relapse of MS, and these are generally avoided in routine post-transplant vaccination schedules. However, there is no clear-cut data to support the reactivation of MS or other ADs following aHSCT and therefore CIBMTR-EBMT, IDSA and ECIL recommendations should be followed with a case-by-case discussion with patients [107, 117, 119]. Measurements of specific antibody titres may be helpful in deciding whether to vaccinate or not [117]. A standard-of-care post-transplant routine vaccination programme may be based on IDSA and ECIL guidelines as follows: pneumococcal conjugate vaccine at 3, 4 and 5 months, followed by conjugate HIB, DTP and inactivated polio vaccine at 6, 7 and 8 months and pneumococcal polysaccharide vaccine at 1 year. Later patients who are not on immunosuppressive therapy (e.g. for relapse) should have serology for measles and varicella tested at 24 months and those who are negative should be immunised with two doses of MMR and varicella vaccine at least 4 weeks apart as per routine practice. Patients should also have an annual Influenza vaccine.

### Neurological follow-up and management of disease activity post-transplant

The disease course after aHSCT should be monitored by regular neurological follow-up, with clinical assessments, imaging and immune markers in blood or cerebrospinal fluid (CSF) appropriate to the disease. In MS, NEDA can be assessed based on the clinical assessment and Gd enhanced MRI of brain and/or spine, which is required at regular intervals post-transplant (at 6 months post-transplant and yearly afterwards). Ongoing rehabilitation and other symptomatic care should be provided as appropriate. Currently, there is no consensus about the management of patients who develop disease activity after aHSCT, including re-introduced DMTs and second aHSCT.

Recommendations

- Post-discharge monitoring should be primarily focused on prophylaxis and management of infection in the first 3–6 months after aHSCT. Antibiotic prophylaxis should be given as per centre protocols, but generally oral prophylaxis should cover fungal infections (with an azole) for 3 months and herpes virus (with aciclovir) and pneumocystis infection for a minimum of 6–12 months post-HSCT (level III).
- PCR-based CMV monitoring is recommended during first 100 days post-HSCT and re-activations should be treated according to institutional protocols, similar to allogeneic HSCT practice (level III).
- PCR-based EBV monitoring is recommended during first 100 days post-HSCT and reactivations managed with imaging and LDH, with rituximab considered on an individual basis (level III).
- Immune monitoring of T- and B-cell subsets and immunoglobulin levels/electropheresis is recommended on a 3-monthly basis in the first year and then annually in order to guide infection prophylaxis and detect paraproteinaemia (level II).
- Centres should ensure systems are in place to provide long-term follow-up. Annual simultaneous follow-up consultation of the neurology and HSCT specialists is recommended. If patients are discharged from the transplant centre for medium- and long-term follow-up under the referring neurologist, annual follow-up should be a standard of care and the contact details should be made available to transplant centre data managers and/or the registry (level III).
- Patients who develop recurrence of disease activity after aHSCT should be managed on an individual case basis. In general, assessment of risk:benefit, including cumulative toxicities of new and re-introduced DMTs should a consideration (level III).

## Mechanisms of action

aHSCT is performed with the premise to reconstitute, and ideally re-condition, the immune system towards a self-tolerant state by depleting the autoreactive immunologic memory with high-dose chemotherapy followed by a profound regeneration of a renewed and diverse immune system, i.e. ‘immune reset’ [120–123].

In MS, a range of mechanistic studies post-transplant have shown that the T-cell repertoire, particularly of CD4^+^ T cells, may be almost completely renewed, its diversity increased and that new thymic output of T cells is achieved following aHSCT [124]. The analysis of TCR repertoires by deep sequencing confirms that aHSCT induces the regeneration of circulating T-cell clones, more profoundly in the CD4^+^ T helper cell compartment [125]. Early post-transplant T-cell repertoire diversity is associated with complete clinical responses during the 5-year follow-up [35, 125].

Other studies examined proinflammatory T-cell effector responses specifically, including Th17 cell frequency, the mRNA expression of their master regulator ROR[gamma]t and the production of the inflammatory cytokine IL-17A all decreased post-HSCT [126]. Several additional immune mechanisms that may contribute to the efficacy of aHSCT in MS have include depletion of peripheral blood mucosal-associated invariant T (MAIT) cells, decrease of MS-associated inflammatory micro RNAs (miR-155, miR-142-3p, miR-16), along with increased immune T and NK regulatory cells and increased expression of immune checkpoint receptors and regulatory molecules such as PD-1, CTLA-4, GITR and TGF-b1 [127].

Other neuroinflammatory diseases have not been studied to any significant extent in the context of immune reconstitution and further research is warranted. The collection of cellular, serum, plasma and CSF samples at baseline, during the immunosuppression-free remission and at relapse/progression for mechanistic and pathogenetic studies in accordance with regulatory requirements for tissue banking and ADWP guidelines is recommended [110].

Recommendations

- Systems for biobanking should be developed alongside clinical trials, routine treatments and registry data in order to support mechanistic and pathogenetic studies in MS and neuroinflammatory diseases (level III).

## Developmental indications: allogeneic HSCT and cell therapy in immune-mediated neurological diseases

### Allogeneic HSCT

Allogeneic HSCT represents an attractive option for patients with refractory ADs, offering the advantage of complete eradication of autoreactive cells combined with the regeneration of a healthy immune system tolerant to autoantigens. However, because of its significantly higher level of NRM risk, allo-HSCT has rarely been used in the treatment of ADs [21, 24, 26, 128]. Only anecdotal data are available to date for allo-HSCT in neuroinflammatory ADs, notably severe NMO, where sustained clinical benefit with resolution of detectable anti-AQP-4Ab has been reported [129].

Major changes have occurred in the field of allo-HSCT [130, 131] including targetted reduced-intensity conditioning and post-transplant tolerising regimens, improved patient and donor selection and better supportive care open up the use of alloHSCT in ADs. Further clinical studies with these modern approaches are warranted.

Recommendations

- Centres performing allogeneic HSCT should have appropriate experience and JACIE accreditation or equivalent (level II).
- Allogeneic HSCT for immune-mediated neurological diseases is developmental and ideally should be performed in a prospective clinical study (level III).
- In the absence of data, conditioning regimens and other allogeneic HSCT technique should reflect the practice in other non-malignant diseases (level III).

### Mesenchymal Stromal Cells (MSC) and other experimental cellular therapies

A range of pre-clinical data and early phase trials provide support for mesenchymal stromal cells derived from autologous and allogeneic sources as immunomodulators with the potential to neuroprotect and foster remyelination endogenous neurogenesis and differentiation in neural cells [132]. Since 2007, over 15 small studies exploring the feasibility and safety of MSC transplantation in multiple sclerosis have been published [133]. These studies involved differing patient populations, cell products and routes of administration. All were underpowered for drawing conclusions on efficacy but reported an overall favourable safety profile. The results of two more similar studies (ACTiMuS, SIAMMS-II) are awaited and a larger randomised, double blind, cross-over phase I/II clinical trial (MESEMS) is ongoing [134–136]. Haematopoietic stem cells genetically manipulated to induce self-tolerance against myelin epitopes have also been explored [137], which may have potential at improving long term remissions following aHSCT. Non-HSCT cell therapies for ADs should be considered a developmental indication as there limited evidence to support administration outside a clinical trial. Generally, there is a need to safeguard vulnerable patients against unjustified hope whilst promoting further clinical trials and basic research [28]. Centres should be accredited according to appropriate JACIE standards relating to immune effector cell (IEC) therapies [86].

Recommendations

- Routine treatment with MSC and other cell therapy is not recommended as there is insufficient evidence as to safety and efficacy in both the inflammatory and progressive phases of MS (level III).
- Patients with MS and other immune-mediated neurological disorders should only be treated with MSCs in clinical trials. Centres should be accredited according to appropriate JACIE standards relating to immune effector cell therapies (level III).

## Future development of HSCT in MS and neuroinflammatory diseases

### Data reporting to the EBMT Registry

Data reporting to the EBMT Registry (and equivalent international registries) has been fundamental to building the knowledge base of HSCT in AD and providing the basis for prospective studies [21, 26]. A major upgrade of the EBMT Registry across all indications is centred around a mandatory core dataset maximising capture of essential data defining the patient, procedure, disease, risks and donor (if relevant), key time points and events required for risk stratification and benchmarking of outcomes. Alongside the core dataset, a modular system is available for defined projects attempting to address strategic research questions generated by the EBMT scientific council, working parties or other working groups. Modules can be used for retrospective data or prospective non-interventional studies. In addition, developments should facilitate the incorporation of non-HSCT treatments with the potential for direct data reporting from neurologists and other disease specialists. All of these aspects are especially relevant for HSCT in MS and other immune-related neurological diseases diseases where the timelines for development of clinical manifestations, particularly evolution of disability, are often long, and evaluation of late effects may take many years.

Transplant centre data managers are generally less familiar with ADs, and many patients are seen in departments outside the transplant centre. Complete data registration has proven more challenging for ADs than standard haematological and oncological indications for HSCT. Data managers should be adequately trained and supervised by relevant HSCT and neurological specialists and ideally neurological data reporting should be integrated by the referring neurologist and their teams. If aHSCT is to be integrated into neurological care pathways, it is vital that efficacy and safety are monitored as robustly as possible via HSCT centres or collaborating neurologists over the long-term. Aligning clinical databases with biobanked samples will allow greater understanding of mechanisms of action and improved risk stratification of patients.

Recommendations

- Data relating to HSCT in MS and other neuroinflammatory diseases should be routinely reported to EBMT or equivalent registry (level III).
- Data managers should be adequately trained and supervised by relevant HSCT and neurological specialists (level III).
- Systems for biobanking should be developed alongside clinical trials and registry data (level III).

### Statistical considerations for clinical studies

Statistical approaches commonly used in other areas of HSCT practice are less easily applied to prospective clinical trials and retrospective studies in MS, where it is important to define appropriate target endpoints to assess the response to administered treatments, whether they are HSCT or other potent treatments. Fortunately, overall survival (OS) is high and all-cause mortality (including NRM) is rare following aHSCT. However, concepts of NRM, PFS and OS are commonly used in HSCT but are unusual to neurologists. Moreover, relapses independent of disease progression do not always represent a treatment failure. Progression of disability can be related to an advanced stage of the disease at HSCT and should not be considered as a treatment failure if not associated with recurrence of neuroinflammation.

Given the growing evidence that an early therapy escalation in aggressive forms may prevent both the development of severe disability and the shift towards the progressive phase through the permanent abrogation of inflammatory activity in the CNS, a reliable assessment of treatment response must include both clinical and radiological metrics, as combined in ‘NEDA’ status [8]. Rate of NEDA in a set of patients at a given time from the treatment start and/or time to maintain a NEDA status are currently considered the most reliable assessment of treatment efficacy in MS and should be considered in any HSCT trial [39, 40]. Improvement in EDSS is an endpoint that has been increasingly used for aggressive therapies in MS and should be included among the endpoints to assess aHSCT, taking into account not only the magnitude of improvement levels but also its durability.

In addition, validated health-related quality-of-life and neuropsychological instruments are important and easily achievable endpoints. Brain volume loss, optical coherence tomography (OCT), corneal confocal microscopy and PET imaging may increasingly provide more sophisticated means of quantifying efficacy in the clinical trial setting. Alongside efficacy, there is the question of the risks of late effects of aHSCT compared with modern DMTs, several of which may have been administered to patients prior to transplant.

RCTs are the best means to establish the safety and efficacy of aHSCT versus alternative ‘standard of care’. Although this approach may be feasible for aHSCT in MS, there will always be the challenge of ‘standard of care’ evolving as new DMTs emerge, especially if recruitment is slow. This was an issue in the MIST trial, where alemtuzumab became a standard of care in the years taken to complete recruitment for the trial [41, 138] and now ocrelizumab and cladribine currently provide similar competition for ongoing studies. In the rarer immune-mediated neurological disease indications RCTs are unlikely to be feasible, and other clinical trial designs may be more appropriate and ongoing retrospective studies and prospective non-interventional studies based around the EBMT registry (which is generally limited to patients receiving HSCT, making comparison with standard of care difficult) along with other neurologically based registries, such as MSBase, may provide meaningful clinical data via prospective cohort studies and case-control studies. The recognition of potential bias and adjustment for all potential prognostic factors is essential in any non-randomised setting in order to accommodate inevitable confounding factors and selection bias in choosing aHSCT over another treatment.

Recommendations

- Where feasible, HSCT for MS and other immune-mediated neurological diseases should be offered in a clinical trial (level III).
- In any study of MS and other immune-mediated neurological diseases, well-defined and validated parameters should be used to define response, progression and remission. For MS, the NEDA status is appropriate for this purpose and feasibly collected alongside other transplant data in the EBMT Registry (level III).
- Magnitude and durability of EDSS improvement should be included as an endpoint for evaluating aHSCT in MS (level III).
- Prospective non-interventional studies provide an alternative and pragmatic means of increasing clinical knowledge, while eliminating bias associated with retrospective studies (level III).
- Although prospective studies are preferred, significant challenges should be recognised in their application to HSCT especially in the rare immune-mediated neurological diseases. When clinical trials are not available then patient data should be sent to EBMT (or equivalent) registry (level III).

#### Clinical trials of aHSCT in MS

While it is now clear that clinical and MRI activity in patients with highly active RRMS may be suppressed with the use of aHSCT in a sustained manner, there remains a need for comparative studies that randomise patients to aHSCT versus other high-efficacy therapies, particularly the more recently introduced alemtuzumab, ocrelizumab and cladribine, where there are highly relevant research questions regarding relative reported rates of NEDA, albeit across prospective trials in RR-MS with varying eligibility criteria, as summarised in Table 4. Current clinical trials, designed with a view to answer these and other questions, are summarised in Table 5.

**Table 4:** Mechanism of action and the relative rates of NEDA in prospective trials of high efficacy DMTs and autologous HSCT in RRMS

Therapeutic	Mechanism of action	Rate of NEDA	Ref
Alemtuzumab	Anti-CD52 monoclonal antibody	39–32% at 2 years	[44, 138, 142]
Ocrelizumab	Anti-CD20 monoclonal antibody	48% at 96 weeks	[143]
Cladribine	Synthetic deoxyadenosine analogue	47% at 96 weeks	[144]
Autologous HSCT with intermediate-intensity conditioning	Immune ablation and reconstitution Cy-ATG (with unmanipulated graft)	93.3%, median follow up 2 years	[33]
	BEAM-ATG (with CD34+ selected graft)	69.2% (EFS), median follow up 5 years	[35]

The trials differ in eligibility criteria and design, including prior DMT treatment and disease activity at study entry. The reader is referred to the original publications for more detailed comparison

**Table 5:** Currently active clinical trials of autologous HSCT in MS

Trial/identifier	Description	Centres/countries
RAM-MS NCT03477500	Phase III RCT of autologous HSCT (Cy-ATG) versus alemtuzumab (later extended to ocrelizumab and cladribine)	Scandanavia, Netherlands
STAR-MS	Phase III RCT of autologous HSCT (Cy-ATG) versus alemtuzumab or ocrelizumab	UK
BEAT-MS	Phase III RCT of autologous HSCT (BEAM-ATG) versus standard of care	US predominantly (NIH-led)
MOST NCT03342638	Phase III RCT of autologous HSCT regimen (Cy-ATG versus Cy-ATG + intravenous immunoglobulin)	Northwestern University, US
COAST	Phase II RCT of autologous HSCT (Cy-ATG) versus ocrelizumab or alemtuzumab	Germany
NET-MS (Italian collaborative)	Phase II RCTof autologous HSCT (BEAM-ATG) versus best available DMT	Italy
Swiss aHSCT Registry Study	Open study of autologous hematopoietic stem cell transplantation in patients with RRMS and progressive forms of MS (5 year duration)	University Hospital Zurich, Switzerland
Mexican open label study NCT02674217	Outpatient Hematopoietic Grafting in Patients With Multiple Sclerosis Employing Autologous Non-cryopreserved Peripheral Blood Stem Cells: A Feasibility Study	Clinica Ruiz, Puebla, Mexico

Another question is to whether aHSCT may offer benefit for the progressive forms of MS, which may continue to have elements of ongoing and resistant neuroinflammation. In the last two decades, a large number of patients with progressive disease have been treated with aHSCT and there is some evidence for reduced relapse rates and clinical stabilisation, but it is difficult to interpret these studies due to the lack of control groups [15, 16, 20, 31, 38, 47]. Further RCTs are required to assess the therapeutic benefit of aHSCT in SPMS and PPMS with evidence of significant inflammation.

### Public health system delivery of HSCT in MS and immune-mediated neurological diseases

At a public health level, economic evaluation is a central consideration in delivering aHSCT for MS and neuroinflammatry diseases. MS results in a large burden on both the health and social care systems as well as the wider exchequer. The costs incurred range from direct costs related to treatment with DMTs, but also reduces long-term quality of life and leads to unemployment, progressive disability and eventually dependency, high rates of unemployment with substantial impact on the affected individual and their carers with reduced quality of life and on the health care service. Compared with ongoing repeated treatments with modern DMTs, aHSCT is a ‘one-off’ treatment, for which, therapeutic benefits last for many years in appropriately selected patients. Favourable cost-effectiveness ratio in MS patients showing a sustained response to HSCT over some DMTs has been reported [139–141]. However, for accurate and up-to-date evaluations, health economic evaluation should be combined with prospective clinical trials. There is great variability in funding for aHSCT in MS and other ADs across EBMT countries, and further evaluations are needed to provide equitable access according to clinical benefit as close to patients’ homes as feasible.

Recommendations

- Health economic evaluations are central to informing the effective delivery of HSCT for MS and other neurological disorders across various health services (level III).
- Engagement with public health authorities and other payers is essential across health services, enabling treatment and coordination of early- and long-term follow up as close to patients’ homes as feasible (level III).

## Conclusions

We have reviewed the evidence for aHSCT for a range of immune-mediated neurological diseases which may respond to aHSCT when other standard treatments have failed, or are deemed likely to fail because of poor-prognostic features. The evidence for effectiveness is highest in highly active RRMS where there is growing evidence from large registry studies and a prospective phase III RCT supporting the safe delivery of aHSCT with long-term clinical and MRI remissions observed in a majority of patients (S/I). In progressive MS and other neuroinflammatory indications data are heterogeneous (CO/II) and aHSCT should be delivered on a clinical trial, if available. The evidence for allogeneic HSCT is developmental (D/III). There is a need for clinical trials across all settings.

Close co-operation between HSCT and neurological specialists in MS and neuroinflammation is critical. In addition to EBMT and national societies, the support of national and international MS and neurological societies is also essential to achieve education, and ultimately acceptance and implementation of this one-off intensive approach to MS and other immune-related neurological diseases. Patient groups, such as the EBMT Patient Advocacy Committee and national MS and other patient associations are also important. Centres of specialisation and experience will be required to support others in bringing HSCT appropriately into neurological clinical practice alongside modern DMTs. Standardisation of practice will assist the support that experienced units can provide to less experienced units. At a public health level, health economic evaluations will be necessary to support decision making and optimise equitable access to evidence-based treatments in publically-funded and private healthcare systems [21, 28].
